# Outcomes and prognostic factors of infantile acquired hydrocephalus: a single-center experience

**DOI:** 10.1186/s12887-023-04034-w

**Published:** 2023-05-24

**Authors:** Faliang Zhou, Zhao Yang, Zezhong Tang, Yang Zhang, Hongmei Wang, Guoyu Sun, Rui Zhang, Yi Jiang, Congle Zhou, Xinlin Hou, Lili Liu

**Affiliations:** 1 Department of Pediatrics, Peking University First Hospital, No.1 courtyard, Xi’anmen Street, Xicheng District, Beijing, China; 2 Office of Academic Research, Peking University First Hospital, Beijing, China; 3 Department of Neurosurgery, Peking University First Hospital, Beijing, China

**Keywords:** Adverse outcomes, Infantile hydrocephalus, Prognosis, Prediction

## Abstract

**Aim:**

To assess the etiologies and adverse outcomes of infantile acquired hydrocephalus and predict prognosis.

**Methods:**

A total of 129 infants diagnosed with acquired hydrocephalus were recruited from 2008 to 2021. Adverse outcomes included death and significant neurodevelopmental impairment which was defined as Bayley Scales of Infant and Toddler Development III score < 70, cerebral palsy, visual or hearing impairment, and epilepsy. Chi-squared was used to evaluate the prognostic factors of adverse outcomes. A receiver operating characteristic curve was calculated to determine the cutoff value.

**Results:**

Of 113 patients with outcome data, 55 patients (48.7%) had adverse outcomes. Late surgical intervention time (13 days) and severe ventricular dilation were associated with adverse outcomes. The combination of surgical intervention time and cranial ultrasonography (cUS) indices was a better predictive marker compared with any of them (surgical intervention time, P = 0.05; cUS indices, P = 0.002). Post-hemorrhage (54/113, 48%), post-meningitis (28/113, 25%), and hydrocephalus arising from both hemorrhage and meningitis (17/113, 15%) accounted for a large proportion of the etiologies in our study. Hydrocephalus occurs secondary to post-hemorrhage and had a favorable outcome compared with other etiologies in both preterm and term groups. A significant difference in adverse outcomes between the inherited error of metabolism as a cause and other etiologies (P = 0.02).

**Conclusion:**

Late surgical treatment times and severe ventricular dilation can predict adverse outcomes in infants with acquired hydrocephalus. It is crucial to identify the causes of acquired hydrocephalus to predict the adverse outcomes. Research into measures of improving adverse outcomes following infantile acquired hydrocephalus is urgently necessary.

## Introduction

Infantile hydrocephalus is a life-threatening condition resulting from excess cerebrospinal fluid accumulation in the ventricles of the brain. At a rate of approximately 0.7-1 every 1,000 live births, infantile hydrocephalus is not uncommon [1, 2]. In infants, hydrocephalus without an obvious extrinsic cause is referred to as congenital hydrocephalus [3]. Hydrocephalus occurring as a complication of another condition is called acquired hydrocephalus. The predominant forms of acquired hydrocephalus are posthemorrhagic hydrocephalus (PHH), which comprises approximately half of all neonatal hydrocephalus cases, followed by postinfectious hydrocephalus, and tumor-related hydrocephalus [4]. Neurodevelopmental outcomes in terms of social and neurocognitive function in congenital hydrocephalus have been reported [5, 6]. Although many studies have evaluated the long-term outcomes and short-term follow-up of post-hemorrhagic hydrocephalus (PHH) and postinfectious hydrocephalus for preterm neonates, [7–9] other etiologies and their prognostic predictors are rarely mentioned. Few studies have discussed the prognostic factors in predicting the neurodevelopmental outcomes of acquired infantile hydrocephalus [10].

Our study aimed to investigate the different causes of infantile hydrocephalus and determine the adverse outcomes and prognostic factors in infants (18 months) at our center, which is a referral center for hydrocephalus in northern China.

## Materials and methods

### Study design

This study conducted a retrospective analysis from the Peking University First Hospital between January 1st, 2008, and January 1st, 2021. Informed consent was obtained from the parents or legal guardians to participate in the study. Ethics approval for this study was granted by the Medical Ethics Committee of Peking University First Hospital.

Enrollment criteria were: (1) Hydrocephalus diagnosed based on clinical findings (large head, bulging anterior fontanel, suture separation, sunset phenomenon of the eyes, bradycardia, or hypotonia) and radiological features. The diagnostic criteria for infantile hydrocephalus are defined as the ventricular index (VI) > 97th centile. The following measurement indices were used for comprehensive evaluation [11]: anterior horn width > 4 mm, thalamo-occipital distance (TOD) > 26 mm and third ventricular width (TVW) > 3 mm (all > 1 mm over the 97th centile). Ventricular height (VH) (> 15 mm) was also measured because it was crucial for enhanced sensitivity to ventricular size, independently of subependymal hemorrhage in the anterior and occipital horns. (2) All patients performed routine cerebrospinal and/or ventricular fluid testing, and urine gas chromatography mass spectrometry and blood liquid chromatography-tandem mass spectrometry for detecting metabolic diseases. (3) Complete regular follow-up until 18 months or die within 18 months. Exclusion criteria were: severe perinatal brain injury from hypoxic-ischemic encephalopathy, brain damage followed by hypoglycemia, bilirubin encephalopathy, etc.

Meningitis, as an etiology, was represented as proven meningitis and suspected meningitis. Proven meningitis was defined as the detection of bacteria from cerebrospinal fluid (CSF) by culture. Suspected meningitis was defined as a negative CSF culture with all the following clinical signs and symptoms of meningitis (temperature instabilities, lethargy, respiratory distress, apnea, jaundice), and changes in the cerebrospinal fluid (CSF pleocytosis (> 30 × 10^6^/L), lower initial CSF glucose (< 1.1mmol/L) and higher protein (> 2 g/L)).

### Hydrocephalus management

Infants meeting enrollment criteria were monitored using cranial ultrasonography (cUS) and daily clinical assessments of the head circumference and anterior fontanel. Surgical intervention time is defined as the period of time from hydrocephalus diagnosis to the date of surgical intervention at our center.

Temporizing neurosurgical procedures (TNPs) were performed upon the surgeon’s discretion depending on whether the head circumference and cUS indices increased despite serial LPs or for VI over P97 + 4 mm [12]. The TNPs involved ventricular access devices or an Ommaya reservoir, external ventricular drainage (EVD), and ventriculosubgaleal shunts. The temporary measures aimed at reducing ventricular dilation by draining or aspirating 10–20 ml/kg of cerebral spinal fluid (CSF) daily, by continuous cUS monitoring.

**Fig. 1:**
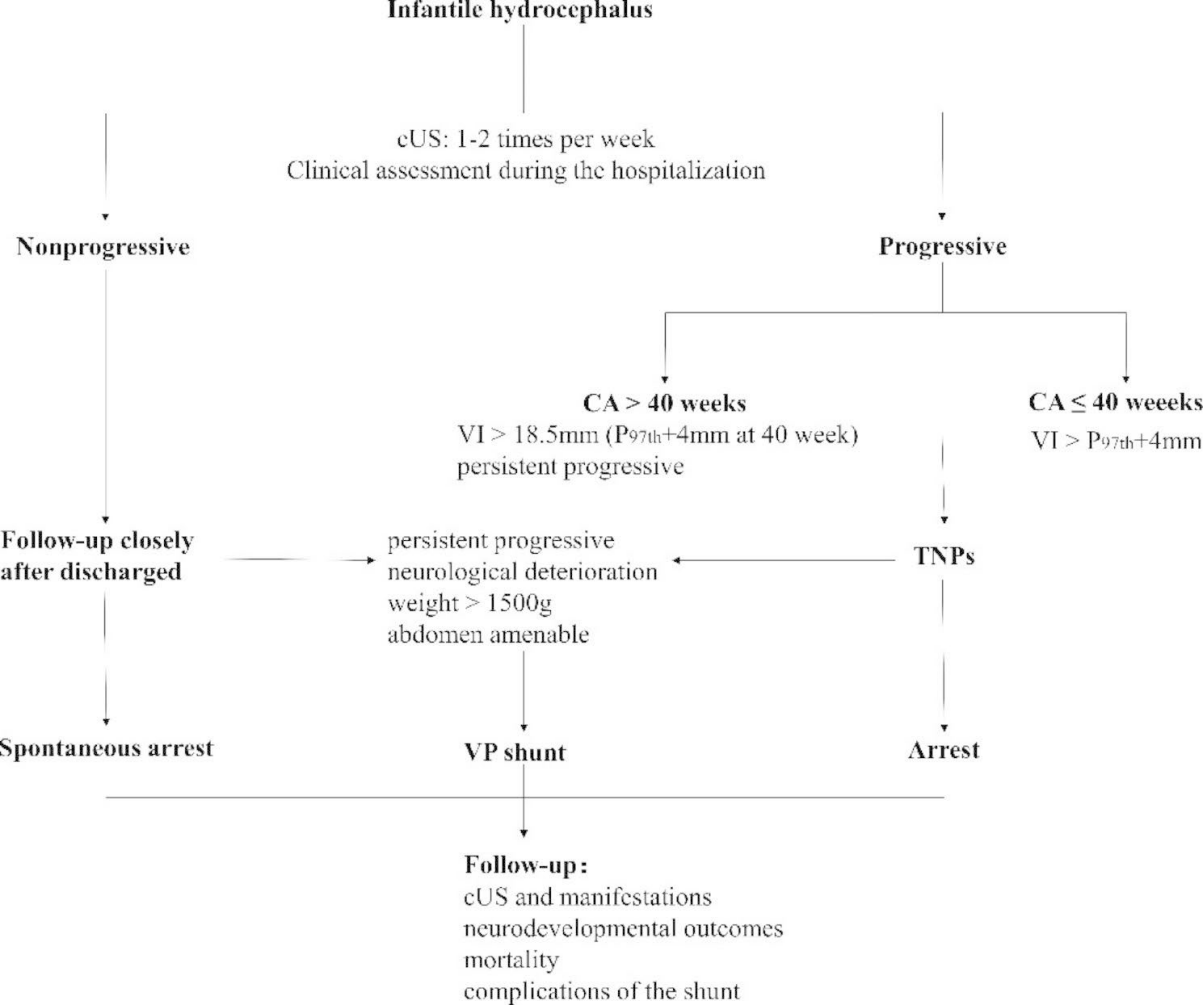
Flowchart of infantile hydrocephalus management Definition of nonprogressive hydrocephalus: (1) the measurement indices are stable or decreased upon the latest continuous cranial ultrasonography (cUS) and (2) clinical manifestations include a large head, bulging anterior fontanel, suture separation, irritability, sunset phenomena of the eyes, and hypotonia, without neurological deterioration. Temporizing neurosurgical procedures (TNPs) were performed upon the surgeon’s discretion if the head circumference and cUS measurement indices increased. The surgical intervention time was according to the reference ventricular index (VI) value for the corrected age at hydrocephalus onset less than or equal to 40 weeks [30]. The VI greater than 18.5 mm (P97th + 4 mm at 40 week) or persistent progressive cUS indices with neurological deterioration were the surgical intervention time for the patients at onset corrected age greater than 40 weeks. TNPs include ventricular access devices or Ommaya reservoir, external ventricular drainage, and ventriculosubgaleal shunts. The appropriate technique was selected based on the surgeon’s discretion. The TNPs aimed at reducing ventricular dilation by draining or aspirating 10–20 ml/kg of cerebrospinal fluid daily as per continuous cUS monitoring and clinical assessment. Moreover, it is crucial to evaluate intraoperative and postoperative complications. Abbreviation: cUS, cranial ultrasonography; VP, ventriculoperitoneal; VI, ventricular index; CA, corrected age

The patients were treated with individualized temporizing measures to drain the CSF before permanent diversion via a ventriculoperitoneal shunt (VPS). VPS was offered in cases of continuous progressive hydrocephalus, such as those characterized by neurological deterioration, increased head circumference, and increased measurement indices on successive cUS under TNPs [13]. A detailed flowchart of infantile hydrocephalus management was shown (Fig. 1). All cUS was performed by an experienced technician following the guidelines of neurosonography in neonates and infants.

### Clinical outcomes at corrected age of 18 months

Patient follow-up evaluations included physical examination (including head circumference, body length, and weight) and cranial ultrasound scans (performed by two professional neonatologists at a corrected age of 3, 6, 9, 12, and 18 months). All hydrocephalus infants evaluated in our center are subjected to a preventive management approach, as they are considered to have a high risk in terms of neurodevelopmental problems. Professional rehabilitation physicians provide a thorough evaluation and intervention (physiotherapy and infant stimulation of early intervention, teaching the parents exercises with the baby at home) for these infants during a stable period of the disease. The rehabilitation planned for infantile hydrocephalus would be an individual program of the best choice. Surviving infants were evaluated using the Bayley Scales of Infant and Toddler Development III (BSID-III), by certified examiners who were blinded to neonatal clinical variables during the follow-up period. BSID-III includes cognitive, language, and motor subscales and a score of < 70 represented a performance of 2SD or more below the mean [14].

A diagnosis of cerebral palsy is usually made between 12 and 24 months of age when there were clinical findings of impaired movement, posture, or balance, and the impairment is permanent and nonprogressive. Hearing impairment was defined as permanent hearing loss affecting language acquisition, education, and overall wellbeing. Visual impairment was defined as visual acuity less than 6/12 in better eyes [15]. Epilepsy is characterized by an enduring predisposition for epileptic seizures, diagnosed by electroencephalography (EEG) or neuroimaging.

Significant neurodevelopmental impairment (sNDI) was defined by having one or more of the following: a BSID score of < 70, epilepsy, cerebral palsy, and visual or hearing impairment.

### Statistical analysis

Statistical analysis was performed using SPSS Statistics version 26.0 (SPSS Inc., Chicago, Illinois, USA) and MedCalc version 20.0.3 (MedCalc Software Ltd., Belgium). Continuous variables (such as age, birth weight, and CSF value) with normal distribution are presented as mean ± SD. Categorical variables are presented as frequency (percentage) and were compared using Pearson’s chi-squared or Fisher’s exact test for small samples. Significance was set at p < 0.05. A receiver operating characteristic (ROC) curve was calculated to determine the cutoff value (maximizing Younden’s index) of the measurement indices in cUS and the earliest treatment time.

## Results

**Fig. 2:**
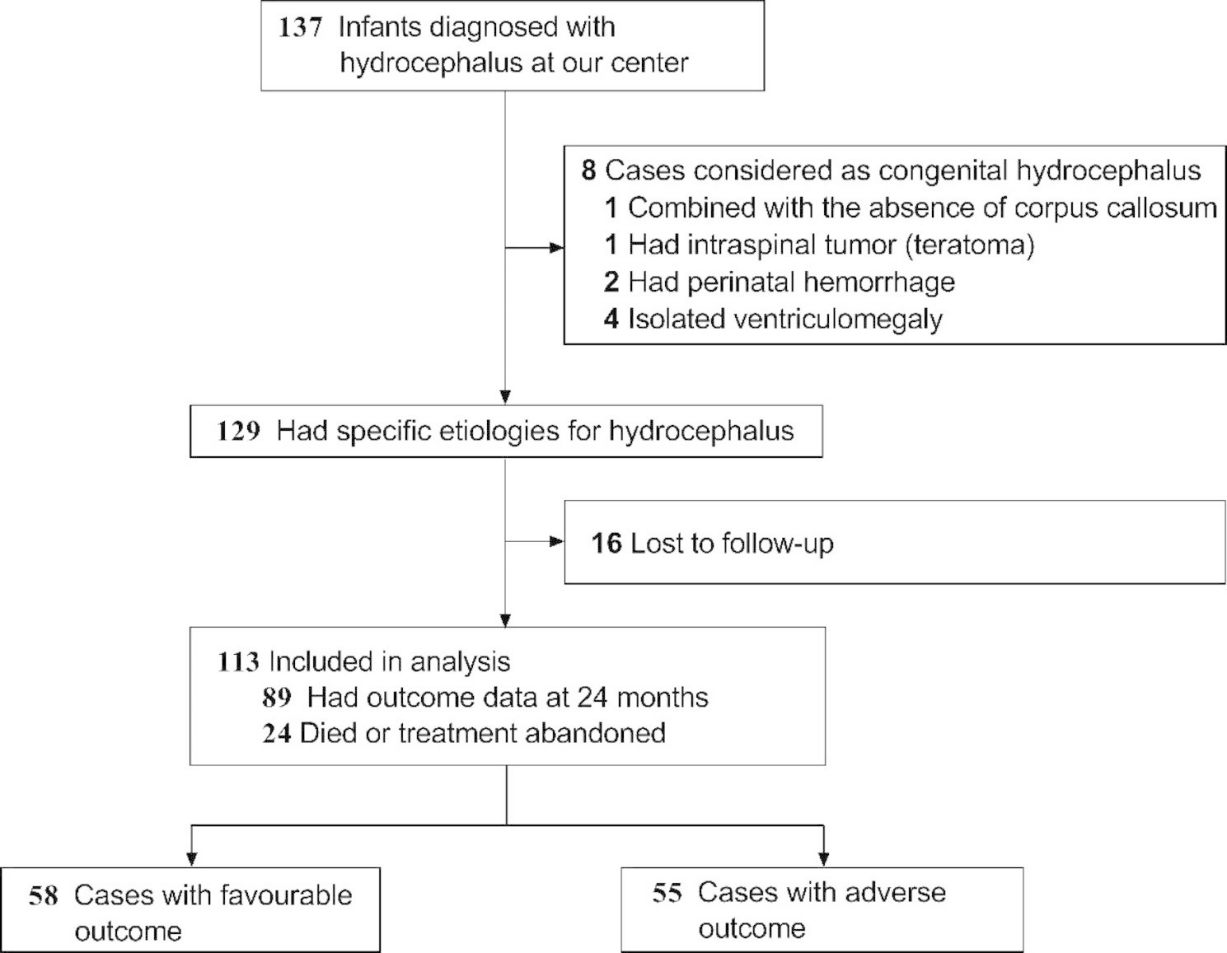
Flow diagram of study participants

Over the 12 years, 137 infants were diagnosed with hydrocephalus at our hospital. Among these, 8 were considered to have congenital hydrocephalus and 16 were lost to follow-up at the time of data collection. Finally, 113 patients with outcome data were included in the analysis (Fig. 2). Of 113 cases, 55 patients (48.7%) had adverse outcomes (sNDI and death), and 58 (51.3%) did not. The baseline characteristics of the study group cases are shown in Table 1.

**Table 1:** Baseline characteristics of the 113 cases

Characteristics	n = 113 (%)
Maternal	
Mother’s age (y)	29.8 ± 5.2
High school graduate	73 (64.6)
Parity	
1	54 (47.8)
2	26 (23.0)
3	17 (15.0)
>3	14 (12.4)
Multiple pregnancy (twins)	17 (15.0)
Diabetes	14 (12.4)
Hypertension	11 (9.7)
Abnormal thyroid function	4 (3.5)
PROM	16 (14.2)
Placental abruption	3 (2.7)
Cesarean delivery	48 (42.5)
**Neonatal**	
Gestational age (wk)	35.5 ± 4.1
Premature infant	59 (52.2)
Birth weight (g)	2,419.1 ± 961.4
Male	75 (66.4)
IVF-ET	3 (2.7)
Intrauterine distress	17 (15.0)
Asphyxia	18 (15.9)

Abbreviations: PROM, premature rupture of membranes; IVF-ET, in vitro fertilization and embryo transfer; CI, confidence interval

The multivariate analysis of the favorable vs. adverse groups was presented in Table 2. Variables including age at presentation, the occurrence of convulsions, and biochemical indicators of CSF were not significantly associated with neurodevelopmental outcomes. 18 cases in our study were considered spontaneous arrests. Of these, 6 patients had sNDI (6/55, 10.9%), and 12 (12/58, 20.7%) did not. 79 cases required TNP as the primary intervention. 33 (42%) patients need further permanent diversion due to the hydrocephalus progression. No significant difference (p = 0.16) in adverse outcomes was found between the two groups. Of the 54 cases of hydrocephalus of post- hemorrhagic etiology, cUS revealed that 48 involved Papile grade 3 hemorrhage and 6 involved grade 4 hemorrhage; grade 4 hemorrhage was associated with poorer outcome (p = 0.036).

**Table 2:** Multivariate analyses associated with clinical outcomes of the 113 patients

	Favorable outcome (n = 58) (%)	Adverse outcome (n = 55) (%)	*p* Value
**Male**	36 (62.1)	39 (70.9)	0.98
**Gestational age (wk)**	35.6 ± 3.9	35.4 ± 4.4	0.79
**Age at presentation (d)**	23.7 ± 22.8	30.0 ± 29.9	0.12
**Etiologies**			
Post hemorrhage	32 (55.2)	22 (40.0)	0.12
Post meningitis	15 (25.9)	13 (23.6)	0.78
Both post hemorrhage and post meningitis	8 (13.8)	9 (16.4)	0.70
Inherited error of metabolism	1 (1.7)	10 (18.2)	
Tumor/cyst/mass lesions	2 (3.4)	1 (1.8)	
**Surgical intervention time (d)** ^a^	12.5 ± 9.9	33.7 ± 38.1	0.002
Ommaya reservoir	29 (50.0)	18 (32.7)	
EVD	11 (18.9)	13 (23.6)	
Subgaleal shunt	3 (5.1)	3 (5.5)	
VPS	1 (1.7)	3 (5.5)	
Intracranial cysto-ventricular shunt	2 (3.4)	0	
**Spontaneous arrest**	12 (20.7)	6 (10.9)	0.16
**Convulsion at onset**	17 (29.3)	19 (34.5)	0.36
**Cerebrospinal fluid**			
Glucose (mmol/L)	1.11 ± 0.71	1.15 ± 1.05	0.82
Protein (g/L)	2.73 ± 2.51	2.75 ± 2.43	0.97
Chloride (mmol/L)	116.4 ± 6.4	115.6 ± 7.2	0.54
**Cranial ultrasonogram**			
Papile grade (n = 54) ^b^	32	22	0.036
Grade 3	31 (96.9)	17 (77.3)	
Grade 4	1 (3.1)	5 (22.7)	
Left VI (cm)	2.09 ± 0.54	2.49 ± 0.76	0.002
Right VI (cm)	1.98 ± 0.49	2.42 ± 0.91	< 0.001
Left VH (cm)	1.73 ± 0.66	2.41 ± 1.13	< 0.001
Right VH (cm)	1.65 ± 0.64	2.29 ± 1.17	< 0.001
Left TOD (cm)	2.79 ± 0.84	3.26 ± 0.98	0.007
Right TOD (cm)	2.44 ± 0.76	2.91 ± 0.95	0.001
TVW (cm)	0.90 ± 0.62	1.11 ± 0.47	0.04
**Abnormal in aEEG**	18 (31.0)	23 (41.8)	0.17
**Permanent shunt**			
VP shunt	18 (31.0)	20 (36.4)	0.55
Age at VP shunt (mo)	6.9 ± 3.3	5.5 ± 2.5	0.14

Abbreviations: sLP, serial lumbar puncture; EVD, external ventricular drainage; VP shunt, Ventriculoperitoneal shunt; VI, ventricular index; VH, ventricular height; TOD, thalamo-occipital distance; TVW, third ventricular width; EEG, electroencephalogram; sNDI, Significant Neurodevelopmental Impairment; CI confidence interval

Adverse outcome: death plus sNDI.

^a^ Surgical intervention time is defined as the period of time from hydrocephalus diagnosis to the date of surgical intervention at our center

^b^ 54 cases diagnosed with posthemorrhagic hydrocephalus and had the outcome data during the follow-up. P value is from Fisher exact test

**Fig. 3:**
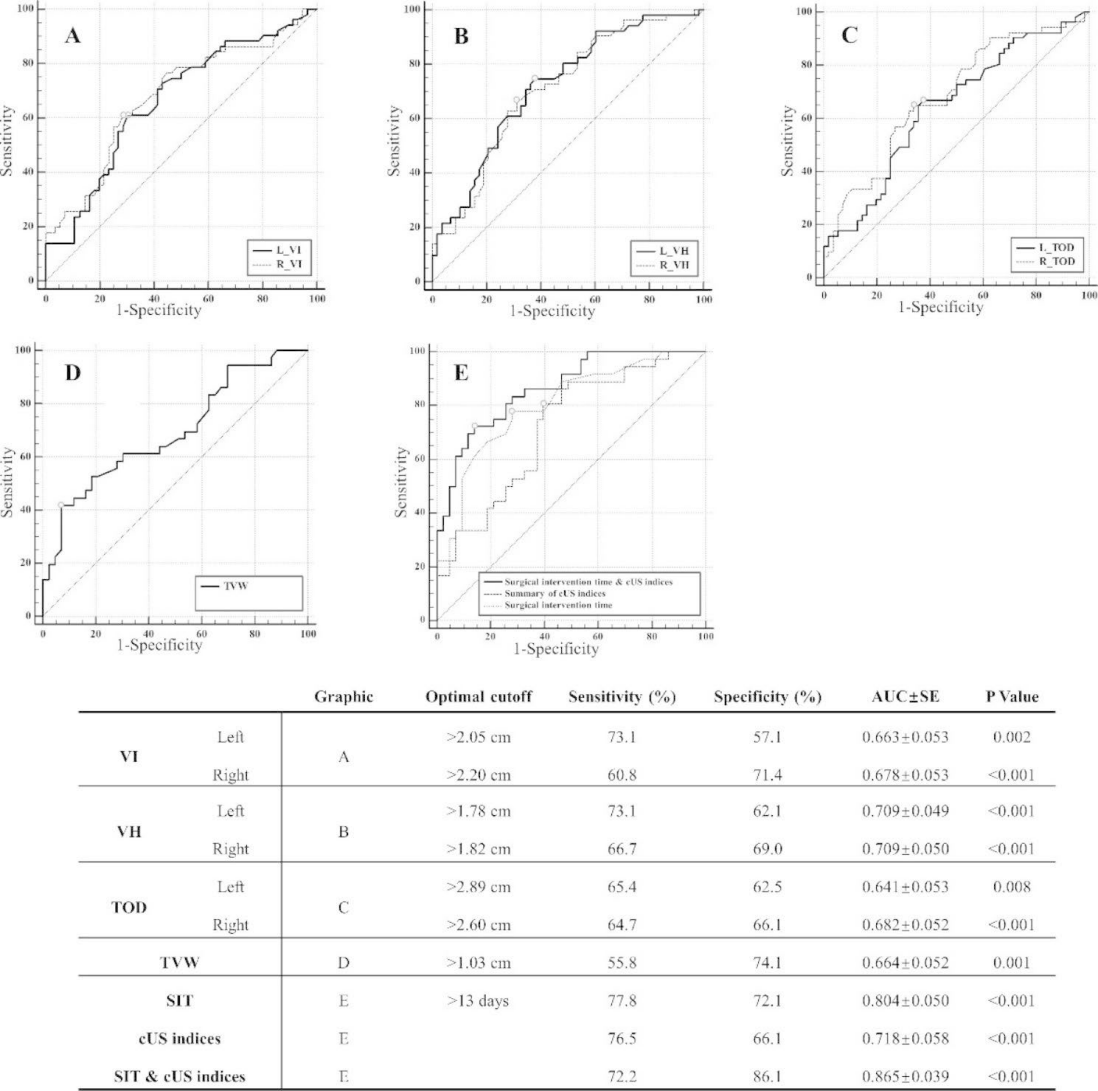
ROC analysis of cranial ultrasound indices and surgical intervention time. Fig. 3. (**A**) (**B**) (**C**) (**D**) Receiver operating characteristics (ROC) curves demonstrating the ability of TVW and both sides of VI, VH, and TOD in predicting the adverse outcomes of significant neurodevelopmental impairment and death. (E) ROC curves demonstrating the ability of surgical intervention time (SIT), summary of cranial ultrasonography (cUS) indices, and SIT & cUS indices in predicting the adverse outcomes. There were significant differences in the area under the curves (AUCs) between measurements of SIT and SIT & cUS indices (P = 0.05), cUS indices and SIT & cUS indices (P = 0.002). Abbreviations: VI, ventricular index; VH, ventricular height; TOD, thalamo-occipital distance; TVW, third ventricular width; AUC, area under the curve; SE, standard error; SIT, surgical intervention time

A significant difference in the surgical intervention time existed between the favorable and unfavorable groups: 12.5 ± 9.9 and 33.7 ± 38.1 days, respectively (p = 0.002). A cutoff point of 13 days was determined for surgical intervention time, with a sensitivity of 0.77 and specificity of 0.72. The differences in TVW and bilateral VI, VH, and TOD on cUS between the two groups were significant (Fig. 3). The mean cutoff points of VI, VH, and TOD were 2.13, 1.80, and 2.75 cm, respectively. The combination of surgical intervention time and cUS indices had a better predictive value compared with any of them (surgical intervention time, P = 0.05; cUS indices, P = 0.002).

The etiologies and outcome analysis of the preterm vs. term groups were shown in Table 3. Post-hemorrhagic (n = 54 of 113; 48%), post-meningitic (n = 28 of 113; 25%) and hydrocephalus arising from both hemorrhage and meningitis (n = 17 of 113; 15%) accounted for a large proportion of the etiologies. Preterm infants acquire hydrocephalus caused by post-hemorrhage (37/59, 62.7%), meningitis (15/59, 25.4%), and both post hemorrhage and meningitis (7/59, 11.9%), and differing from the term cases with various etiologies (post-hemorrhage, 17/54, 31.4%; meningitis, 13/54, 24.1%; both post hemorrhage and meningitis, 10/54, 18.5%; inherited errors of metabolism, 11/54, 20.4%; tumor/cyst/mass lesions, 3/54, 5.6%). No significant difference was found in outcomes between the preterm and term groups. Totally, 31 patients with sNDI were in our study group. Of these, 13 patients with epilepsy (all cases have development delay evaluated by Bayley scales), 4 with hearing impairment (3 of them have cognitive or language impairment), 3 with visual impairment (1 patient has a global developmental delay, 1 has language impairment delay), and 1 case with cerebral palsy.

**Table 3:** Etiologies and outcomes between preterm and term group

	Preterm group (n = 59) (%)	Term group (n = 54) (%)	*p* Value
**Male**	41 (69.5)	34 (63.0)	0.46
**Gestational age**	32.0 ± 3.5	39.3 ± 1.5	< 0.001
**Birth weight**	1950.7 ± 767.3	3415.4 ± 523.2	< 0.001
**Age at presentation**	24.1 ± 22.6	23.3 ± 28.8	0.94
**Etiologies**			
Post hemorrhage	37 (62.7)	17 (31.5)	0.001
Post meningitis	15 (25.4)	13 (24.1)	0.87
Both post hemorrhage and post meningitis	7 (11.9)	10 (18.5)	0.32
Inherited error of metabolism	0	11 (20.4)	
Tumor/cyst/mass lesions	0	3 (5.6)	
**Bayley scale < 70**	15 (25.4)	14 (25.9)	0.95
Cognitive score < 70	12 (20.3)	9 (16.7)	0.62
Language score < 70	11 (18.6)	8 (14.8)	0.59
Motor score < 70	10 (16.9)	7 (13.0)	0.55
**Epilepsy**	7 (11.9)	6 (11.1)	0.90
**Visual impairment**	2 (3.3)	1 (1.9)	
**Hear impairment**	2 (3.3)	2 (3.7)	
**Cerebral palsy** ^**a**^	1 (1.7)	0	
**sNDI**	17 (28.8)	14 (25.9)	0.73
**Death prior to 6 months**	8 (13.6)	8 (14.8)	0.85
**Death prior to 12 months**	9 (15.3)	11 (20.4)	0.47
**Death**	11 (18.6)	13 (24.1)	0.48
**Death or sNDI**	28 (47.5)	27 (50)	0.79
**VP shunt**	18 (33.3)	20 (37.0)	0.46
**Age of VP shunt (mo)**	6.2 ± 3.0	5.8 ± 2.3	0.67

Abbreviations: VP shunt, ventriculoperitoneal shunt; sNDI, significant neurodevelopmental impairment

^a^ A 4-year-old girl was diagnosed with cerebral palsy at 13 months of corrected GA with involuntary movements, unstable gait, and intellectual disabilities upon recent follow-up. Level II classified by GMFCS.

**Fig. 4:**
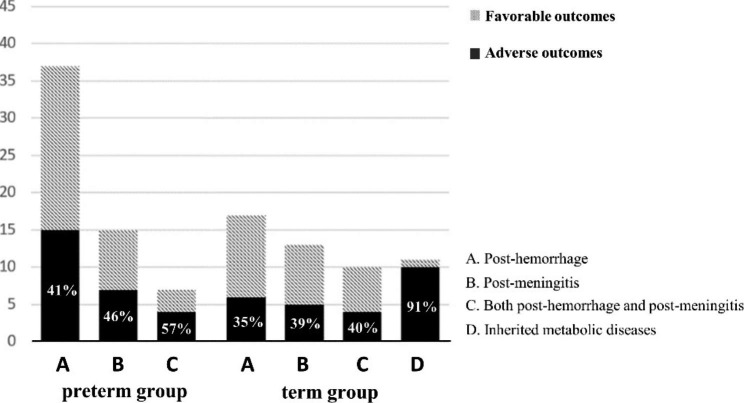
General outcomes of different etiologies Tumor/cyst/mass lesion were rare cases; therefore, they were not counted. Numbers and proportions of the adverse outcomes were shown in the bar chart. The proportions in preterm group of the adverse outcomes among with post-hemorrhagic, post-meningitic, and both post-hemorrhagic and post-meningitic hydrocephalus were 41%, 46%, and 57%, respectively. The proportions of term group in among those with post-hemorrhagic, post-meningitic, both post-hemorrhagic and post-meningitic hydrocephalus, and hydrocephalus from inherited metabolic diseases were 35%, 39%, 40%, and 91%, respectively. Significant difference in adverse outcome proportion between inherited error of metabolism as a cause and other etiologies (P = 0.02).

Figure 4 presents the proportion of adverse outcomes in term and preterm groups by etiology. The proportions in the preterm group of the adverse outcomes in post-hemorrhagic, post-meningitic, and both post-hemorrhagic and post-meningitic hydrocephalus were 41%, 46%, and 57%, which is higher than the term groups 35%, 39%, and 40%.

A total of 45 patients had a post-meningitic etiology, and 22 of these had a proven meningitis, including *Escherichia coli* (n = 10), *Listeria monocytogenes* (n = 4), *Enterococcus Faecium* (n = 3), *Staphylococcus* (n = 2), *Klebsiella pneumoniae*, group B beta-hemolytic *Streptococcus* and *Enterobacter cloacae* (all n = 1). Ten of eleven patients with hydrocephalus from inherited metabolic diseases had poor prognoses (90.9%). Among these, nine patients were diagnosed with methylmalonic aciduria and homocystinuria (MMA-HC, all are cblC type), one was diagnosed with glutaric acidemia type 1, and another patient had an unknown origin with hypertrophic cardiomyopathy, rhabdomyolysis, and liver damage. The mean value of homocysteine in the nine patients with MMA-HC was 120.8 ± 48.9µmol/L. Three patients had tumor/cyst/mass lesions as etiology (arachnoid cyst = 2, intracranial cyst = 1).

## Discussion

Infantile hydrocephalus is a relatively common but severe complication that predicts childhood neurodevelopmental risk. The association between hydrocephalus and poor long-term neurodevelopmental outcomes has been proven, [16] but the analysis of prognostic factors is limited. In this study, we report a general adverse outcomes rate of 48.7%. Late treatment times and ventricular dilation had relatively poor outcomes but were not associated with sex, age at presentation, convulsion at onset, EEG findings, and glucose, protein, and chloride levels in CSF. Besides, the combined measurements of surgical intervention time and cranial ultrasonography indices have a better predictive value for adverse outcome evaluation.

Recent studies have shown that infants with post-hemorrhagic ventricular dilatation require timely treatment for better neurodevelopmental outcomes [13]. These data support the retrospective study of Bassan et al. [17] that suggested early (< 25 days of life) external ventricular drainage (EVD) was associated with low rates of cognitive and communication disabilities than later EVD in infants with post-hemorrhagic hydrocephalus. The timing of drain implantation longer than 1 month after hydrocephalus is diagnosed might be a risk factor for poor mental and linguistic developmental outcomes [18]. However, a cutoff point of 13 days was determined for surgical intervention time in infantile acquired hydrocephalus, with 0.77 sensitivity and 0.72 specificity on ROC analysis. This supports the evidence that infants requiring treatment should be evaluated and operated on promptly on time.

Ventriculomegaly is associated with an increased risk of both cognitive and motor sequelae due to periventricular brain injury [1, 8] This suggests a direct association to prolonged pressure and indirectly, to ischemia and inflammation, leading to damage of white matter from which no recovery capacity exists [19]. A team had succeeded in predicting hydrocephalus outcomes in premature neonates using a cUS-based phenotyping tool [20]. However, these findings are difficult to apply in clinical practice. Herein, we prove that severe ventricular dilation is associated with adverse outcomes, and describe the cutoff point for the VI (2.13 cm), VH (1.80 cm), TOD (2.75 cm), and TVW (1.03 cm).

PHH is a common complication of IVH, and the PHH rate varies between 18% and 35% [21–23]. Adverse outcomes are related to the severity of IVH as the first significant risk factor for PHH. Our results are concordant with findings of reports stating that neurodevelopmental outcomes are worse for grade 4 IVH. Hydrocephalus has been rarely reported in adults with bacterial meningitis, but it is more common in younger children, especially in infants aged < 6 months [24]. 36–63% of patients with bacterial meningitis had a confirmed bacterial etiology [24–26]. In our study, 22 of the 45 patients (49%) with post-meningitic hydrocephalus had a definite pathogen. Of these, 45% reported *E coli* as the pathogenic bacteria, followed by *L.monocytogenes* (7.7%) and *E.faecium* (5.8%). Pediatricians in clinical practice should pay attention to infants who with above positive bacterial cultures for acquired hydrocephalus.

Hydrocephalus has rarely been reported as a sequela of inherited metabolic diseases [1]. MMA is a typical organic acidemia caused by defects in methylmalonyl-coenzyme A mutase or adenosyl-cobalamin synthesis; it is also the most common metabolic cause of hydrocephalus in China [27]. The *MMACHC* gene is the cause of the cblC type, which is the most common subtype always accompanied by homocysteine, of which, the main toxic effect is arterial wall injury; it further reduces the compliance of extracerebral intracranial arteries [27]. Herein, we describe 9 cases of MMA with hydrocephalus, all belonging to the cblC type with homocystinuria (120.8 ± 48.9µmol/L). A Dutch study [28] reported that in a patient with glutaric acidemia type 1 (GA-1) developing ventriculomegaly (9 in 18 individuals with GA-1), characteristically, the acute encephalopathic crisis and movement disorders also occurred. Thus, neurological imaging tests should be performed in patients with metabolic diseases.

Hydrocephalus etiologies were strongly associated with significant neurodevelopmental impairment and mortality. Generally, compared to infants with hydrocephalus caused by other etiologies (infection, congenital malformation, spina bifida), the risk of adverse outcomes was markedly higher in patients with PHH, [10, 29] especially premature infants, which may be due to the ischemic or inflammatory injuries to periventricular brain structure [8, 18]. However, in this study, relatively favorable outcome observed in PHH compared with other etiologies except for the influences of prematurity and hemorrhage combined with central nervous system infection. Infantile acquired hydrocephalus caused by an inherited error of metabolism had a poorer outcome in this limited sample retrospective study and needs to pay more attention.

We are a tertiary referral center, and the sickest hydrocephalic infants from our province and adjoining regions are referred to our hospital. Most cases of delayed surgical intervention were outborn patients because they could not be referred due to severe conditions or refusal from parents, or because hydrocephalus could not be diagnosed early due to lack of regular monitoring. Early detection and intervention (besides surgical procedures) discussed by multiple disciplines with neonatologist, neurosurgeon, physiatrist, and child health doctor was taken for the patients. It likely leads to a better outcome for hydrocephalic infants.

Our study has some limitations. The first is the early endpoint of neurodevelopmental follow-up. Taking the progressive trajectories and longer follow-up of the neurobehavioral development as the child grows is essential for an accurate appraisal. Second, the use of different strategies in individual neurodevelopmental evaluation in the interpretation of outcomes is a limitation of this retrospective study. Finally, heterogeneous etiology was also a limitation of this study. Research into different adverse outcomes of specific etiologies for these patients is urgently required. Previous studies have found that the common causes of acquired hydrocephalus in infants are hemorrhage, neoplasm, and infection, usually bacterial meningitis [1, 3]. The reasons for the difference may be the selection bias of the inclusion criteria for the neonatal units and the high rate of critical disease at our referral center.

## Conclusions

This study provides a comprehensive analysis of the prognostic factors related to infant hydrocephalus, as evaluated at our center over the last 12 years. We found that PHH had a relatively favorable outcome compared with other etiologies except for the influences of prematurity and hemorrhage combined with meningitis. Late surgical intervention time and severe ventricular dilation have relatively adverse outcomes. Further research to reduce the incidence and severity of hydrocephalus in infants and improve their subsequent neurodevelopmental outcomes is urgently needed.

## Data Availability

All data generated or analyzed during this study are included in the submission. The raw data are available from the corresponding author on reasonable request.
